# PTH monitoring after total parathyroidectomy with forearm auto-transplantation: potential for spuriously high levels from grafted forearm

**DOI:** 10.1186/s40463-017-0226-y

**Published:** 2017-06-23

**Authors:** Diana Khalil, Paul D. Kerr

**Affiliations:** Department of Otolaryngology, Health Sciences Center, University of Manitoba, GB421 – 820 Sherbrook Street, Winnipeg, Manitoba R3A1R9 Canada

**Keywords:** Renal failure, Hyperparathyroidism, Total parathyroidectomy, Forearm auto-transplantation, Parathyroid hormone

## Abstract

**Background:**

We have identified a cause of falsely elevated parathyroid hormone (PTH) levels after total parathyroidectomy with forearm auto-transplantation (TPT-ATx). Our cases highlight the need to draw PTH samples remotely, away from forearm graft sites, to ensure accurate levels.

**Case presentations:**

We report on four patients who were referred to our surgical team at an academic tertiary care center for what was perceived to be recurrent hyperparathyroidism 2–5 years following total parathyroidectomy with auto-transplantation. Further evaluation revealed highly discrepant results in these patients depending on where the blood was drawn, with spuriously high levels in blood drawn from the grafted arm (Range 337–3885 ng/l), and much lower levels when blood was drawn remotely away from the graft site (Range 9–242 ng/l). The difference in PTH level between the grafted forearm and remote site for these patients ranged between 328 and 3643 ng/l.

Over the period these cases were accrued (2008–2012), 89 patients underwent TPT-ATx in our institution. Therefore, our case report series suggests that this phenomenon will be evident to a clinically important extent in at least 4% of patients.

**Conclusions:**

One can acquire spuriously high PTH levels from grafted forearms, leading to the false diagnosis of recurrent hyperparathyroidism. We recommend PTH levels be drawn remotely from graft sites to ensure accurate systemic levels are reflected.

## Background

Secondary hyperparathyroidism (SHPT) is a common complication of chronic kidney disease (CKD). Imbalance of calcium and phosphate homeostasis from CKD results in hyperplasia of the parathyroid glands and increased levels of active parathyroid hormone (PTH) [1]. Research indicates that nodular hyperplasia of the parathyroid gland can be found histologically in 75% of CKD patients, with severe, symptomatic SHPT occurring in 5% [2]. SHPT results in a broad spectrum of mineral metabolism disorders, including high bone turnover disease and cardiovascular disease [2, 3]. Although most patients can be treated medically, surgical intervention is recommended in those who suffer from intractable symptoms such as bone pain, pruritus and soft tissue calcification, and in those who develop autonomous function with hypercalcemia [2, 4]. When medical management fails, the surgical options include subtotal parathyroidectomy, and total parathyroidectomy with, or without, forearm auto-transplantation. Many surgeons prefer total parathyroidectomy with auto-transplantation (TPT-ATx).

Postoperatively, patients are monitored for response to treatment and recurrence with serial parathyroid hormone (PTH) levels. We have noted that postoperative monitoring is occasionally confounded by widely fluctuating PTH levels. One possible cause for these fluctuations is the potential for higher levels being drawn upstream from the graft site in the implanted arm. The PTH level in the grafted forearm may be high enough to prompt reassessment for further surgical exploration when in fact the systemic PTH level is at an acceptable level. The patients in this review are examples of this phenomenon, which has not been well described in the literature.

## Case presentations

**Fig. 1:**
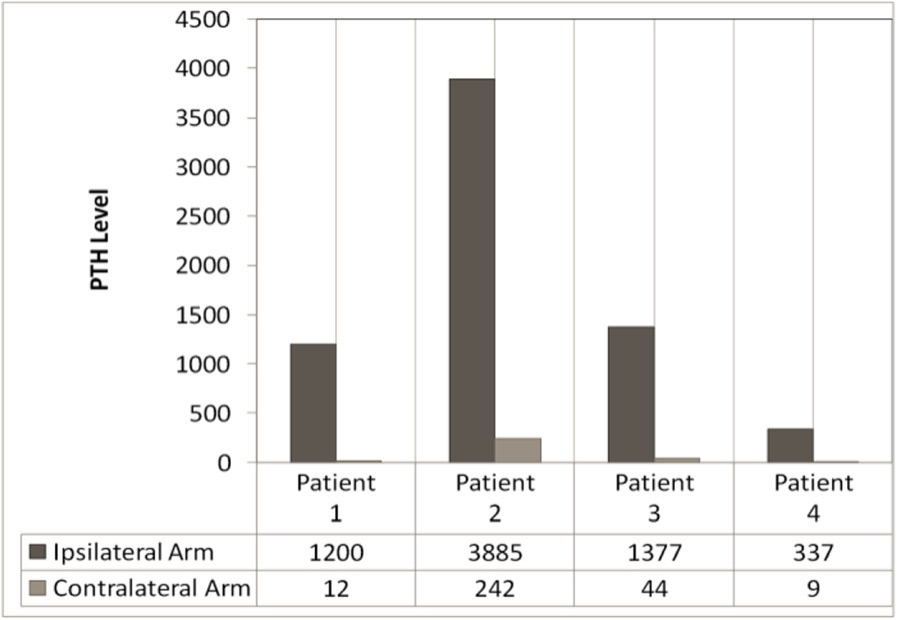
Comparison of post-operative PTH (ng/l) levels drawn from the arm ipsilateral vs contralateral to the parathyroid graft

There were 89 patients that underwent total parathyroidectomy with forearm auto-implantation between January 1, 2008 and December 31, 2012. Four out of these 89 patients (2 males; 2 females) have since been re-referred to our service for what was ultimately determined to be spuriously high PTH levels due to blood samples drawn from the grafted arm. The patients were 2–5 years post TPT-ATx. The spurious results were revealed because our initial workup of recurrent secondary hyperparathyroidism routinely includes a request for confirmation bloodwork from the ungrafted arm, or other site remote from the graft. The highly discrepant results are depicted in Fig. 1. The difference in PTH level between the grafted forearm and the remote site ranged from 328 to 3643 ng/l. All these patients have since been monitored for over 1 year. None of them have required reoperation.

## Discussion

PTH has a very short half-life of approximately 4 min. Given the short half-life of PTH hormone, and the placement of parathyroid grafts in the forearm close to where blood sampling is often performed, we believe that there is significant potential for obtaining erroneously high estimates of serum PTH if the grafted arm is used for venous sampling. Further, we believe that variably sampling the grafted and non-grafted arms, or other sites remote from the graft accounts for some of the variability in postoperative PTH levels that is observed. While this seems self-evident, there are currently no studies in the literature addressing this issue. Failure to account for this phenomenon could easily result in patients undergoing unnecessary testing or even surgery.

The rate of this phenomenon is likely over 4% (4/89). The rate may in fact be higher as this report is simply a series of cases, not a comprehensive longitudinal review of all 89 patients treated over the time that these cases were accrued. Understanding this phenomenon, and noting where blood samples are drawn from, ensures accurate systemic PTH levels are monitored and eliminates the possibility of unnecessary investigations and treatment for recurrent SHPT. Several studies suggest the relative safety and efficacy of total parathyroidectomy without auto-transplantation in the management of renal failure induced hyperparathyroidism [5, 6]. Perhaps the propensity for spurious parathyroid levels after auto-transplantation is another factor worth considering in choosing one procedure over another.

The forearm is frequently used for parathyroid implantation as it is easily accessed during parathyroid surgery, and forearm grafts are easily localized and explored under local anesthetic if they must be removed. In the event of recurrent hyperparathyroidism, it is generally easy to determine if the forearm graft is the cause based on parathyroid scanning, through selective venous sampling, or using a limb ischemia test which should result in substantial reduction of PTH after 15 min of ischemia if the forearm graft is indeed the cause of recurrent disease [7]. There are numerous documented graft locations such as the neck, abdomen, pre-sternal area, or the leg [8–10]. While these locations may be less likely to generate misleading blood test results as they are remote from common blood-drawing locations, there can be disadvantages to these sites such as an inability to perform some of the aforementioned localization and functional testing, more difficult surgical accessibility for removal, concerns regarding leg wound healing in patients with circulatory disease, and cosmesis. The primary disadvantage of the forearm site would appear to be the propensity to cause spurious PTH results. Therefore, one must consider all these issues when choosing an implantation site.

## Conclusions

These cases illustrate how one can acquire spuriously high PTH levels from grafted forearms after TPT-ATx, and thus the potential to falsely diagnose recurrent hyperparathyroidism. We recommend that PTH levels be drawn remotely from graft sites to ensure that accurate systemic levels are acquired.

## Abbreviations

CKD: Chronic kidney disease
PTH: Parathyroid hormone
SHPT: Secondary hyperparathyroidism
TPT-ATx: Total parathyroidecomy with auto-transplantation
